# Anti-Oxidant and Fucoxanthin Contents of Brown Alga Ishimozuku (*Sphaerotrichia divaricata*) from the West Coast of Aomori, Japan

**DOI:** 10.3390/md16080255

**Published:** 2018-07-30

**Authors:** Hayato Maeda, Satoru Fukuda, Hikari Izumi, Naotsune Saga

**Affiliations:** 1 Faculty of Agriculture and Life Science, Hirosaki University, 3 Bunkyo-cho, Hirosaki, Aomori 036-8561, Japan; 2 Institute of Regional Innovation, Hirosaki University, 2-1-1 Yanagawa, Aomori 038-0012, Japan; s.fukuda@hirosaki-u.ac.jp (S.F.); h-izumi@hirosaki-u.ac.jp (H.I.); nsaga@hirosaki-u.ac.jp (N.S.)

**Keywords:** anti-oxidant, fucoxanthin, Ishimozuku, *Sphaerotrichia divaricata*

## Abstract

Fucoxanthin is a specific carotenoid in brown seaweeds with remarkable biological properties. Ishimozuku (*Sphaerotrichia divaricata*), an edible brown alga from northern Japan, has morphology that is almost identical to that of Okinawa-mozuku (*Cladosiphon okamuranus*) harvested off Okinawa, Japan. However, because of Ishimozuku’s lower availability compared to Okinawa-mozuku, the contents of its nutrient compounds remain unclear. The present study analyzed fucoxanthin and anti-oxidant compound contents of Ishimozuku harvested off the northern coast of Japan from 2014 to 2016. First, 80% ethanol extract solutions were prepared from Ishimozuku harvested from several west coast areas of Aomori, Japan. Then, polyphenol content was analyzed using the Folin–Ciocalteu method. Then anti-oxidative effects were analyzed by their 1,1-diphenyl-2-picrylhydrazyl (DPPH) radical scavenging activity and hydrogen peroxide scavenging activity. Furthermore, fucoxanthin contents were measured using high performance liquid chromatography (HPLC) analysis. Fucoxanthin contents of Ishimozuku were 105.6–1148.5 μg/g dry weight. Total polyphenol contents of Ishimozuku were of 0.296–0.958 mg/g dry weight: higher than Okinawa-mozuku (0.082 ± 0.011 mg/g dry weight). The anti-oxidation effects of Ishimozuku accompanied the polyphenol content. These results suggest that Ishimozuku contains various anti-oxidant components and has high potential to provide the promotion of human health.

## 1. Introduction

Various types of algae are included in traditional diets of residents of the Far East and Hawaiian Islands, Japan, Korea, and China. Algae, which contain specific polysaccharides, are used as polysaccharide thickeners and gelling agents for various industrial applications. Recent reports have described that these components show interesting biological activity. Moreover, these water-soluble components have immune system modulation and anti-hypertension activities [1,2]. Polysaccharides in the human body improve the intestinal environment. In addition to that effect, polysaccharides suppress the absorption of extra lipids and cholesterol in the small intestine [3,4,5]. Some of those beneficial components are used today as functional food materials.

Algae lipid fractions contain polyphenols and carotenoids. Many studies have demonstrated that algal polyphenols possess biological activities, including anti-inflammatory, hepatoprotective, anti-tumor, anti-hypertensive, and HIV-1 reverse transcriptase activities as well as anti-diabetic activity based on the inhibition of α-glucosidase [6].

Carotenoids of algae have attracted attention as new functional food ingredients. They are natural pigments contained in many vegetables, fruits, fishes, and algae. Carotenoid types differ among plant species, especially in aquatic plants. The most important biological aspect of carotenoids for humans is the role of a provitamin A activity. Furthermore, dietary carotenoids are known to reduce the risk of cardiovascular diseases, age-related macular degeneration, and cancer [7,8,9,10]. The anti-oxidant properties of carotenoids have been inferred as the main mechanism by which they exert their beneficial health effects [11,12].

Fucoxanthin is a specific carotenoid contained in brown algae, *Undaria pinnatifida* (Japanese Wakame), *Saccharina japonica* (Japanese Makonbu), and *Sargassum fulvellum* (Japanese Hondawara). Actually, fucoxanthin has a unique structure including an unusual allenic bond and 5,6-monoepoxide in its molecule. It has no provitamin A activity, but it shows strong anti-oxidant properties, as do other carotenoids. Furthermore, fucoxanthin was found to be the strongest inducer of apoptosis and anti-proliferation in human cancer cells among 15 investigated dietary carotenoids [13,14]. Fucoxanthin reportedly has anti-tumor properties and prevents carcinogenesis in mice [15,16]. More recently, fucoxanthin has been reported to have anti-obesity, anti-diabetic, and anti-cancer effects [17,18,19,20]. Dietary fucoxanthin induces expression of uncoupling protein 1 (UCP1) and plays an important role in energy expenditure in white adipose tissue. Because of the unique mechanism of these effects, it has attracted much attention in the food industry and nutrition studies.

Ishimozuku (*Sphaerotrichia divaricata*) is an edible brown alga harvested from Hokkaido to Kyushu of Japan. The morphology resembles that of Okinawa-mozuku (*Cladosiphon okamuranus*), but they are different species. In Japan, Okinawa-mozuku and Ishimozuku are edible algae. Most of Okinawa-mozuku is farm-raised in Okinawa prefecture. Especially, Okinawa-mozuku is used in diverse applications as a food material or functional food ingredient because it has strong stickiness. However, Ishimozuku is not such popular algae in markets, probably because the toughness or feel in the mouth of the algal body is somewhat hard compared to those of Okinawa-mozuku.

Ishimozuku is a special product in Aomori, at the far northern end of the main island of Japan. Because of the lower availability of Ishimozuku than that of Okinawa-mozuku, the nutrient compound contents remain unclear. Furthermore, nutrient contents of seafood are normally affected by culture conditions such as seawater contents, depth, water temperature, and season [21,22]. Therefore, for promoting product quality, it is important to evaluate the nutrient characteristics of algae in several harvested areas.

This study evaluated the total polyphenol content, fucoxanthin and anti-oxidative effect of Ishimozuku harvested along the north-west coast of Aomori, Japan, during 2014–2016.

## 2. Results

### 2.1. Amounts of Total Phenolic Compounds

Table 1 shows total phenolic compound contents of Ishimozuku. They were 0.296–0.958 mg/g dry weight. Sample j demonstrated the highest total phenolic compound content (0.958 ± 0.012 mg/g dry weight) whereas sample i demonstrated the lowest (0.296 ± 0.004 mg/g dry weight). Different harvest years did not show a difference in contents. However, the total polyphenol contents of Okinawa-mozuku were low (0.082 ± 0.011 mg/g dry weight).

**Figure 1:**
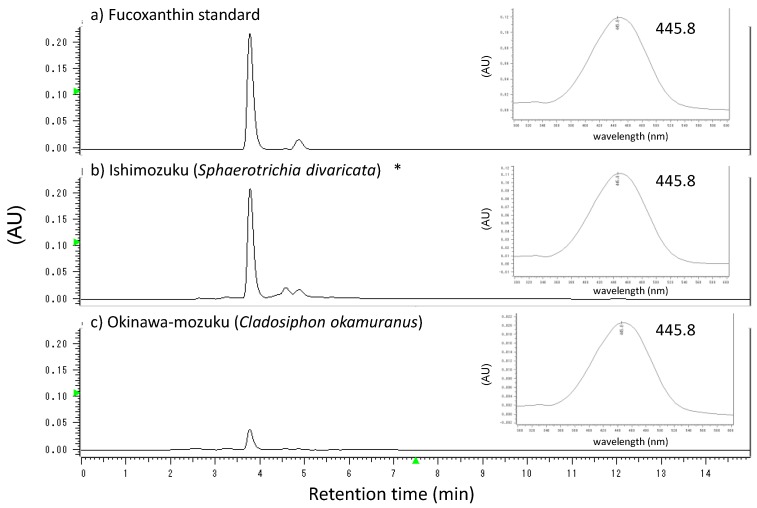
High performance liquid chromatogram (HPLC) of fucoxanthin standard, Ishimozuku extract, and Okinawa-mozuku extract detected at 450 nm. The chromatogram inserted at the upper right is the maximum absorption wavelength at the retention time of fucoxanthin. (* Ishimozuku chromatogram was Sample n.)

Figure 1 shows a high performance liquid chromatogram (HPLC) of fucoxanthin analysis results. Fucoxanthin was detected in the extract of Ishimozuku and Okinawa-mozuku. The maximum absorption wavelength of each peak was consistent with fucoxanthin standard sample. The highest fucoxanthin content was 1148.5 ± 18.9 µg/g dry weight (sample d). The average fucoxanthin content of Ishimozuku was 557.9 ± 93.8 µg/g dry weight. That of Okinawa-mozuku was 153.8 ± 5.80 µg/g dry weight. 

### 2.2. Anti-Oxidative Activity of Ishimozuku Extract 

**Figure 2:**
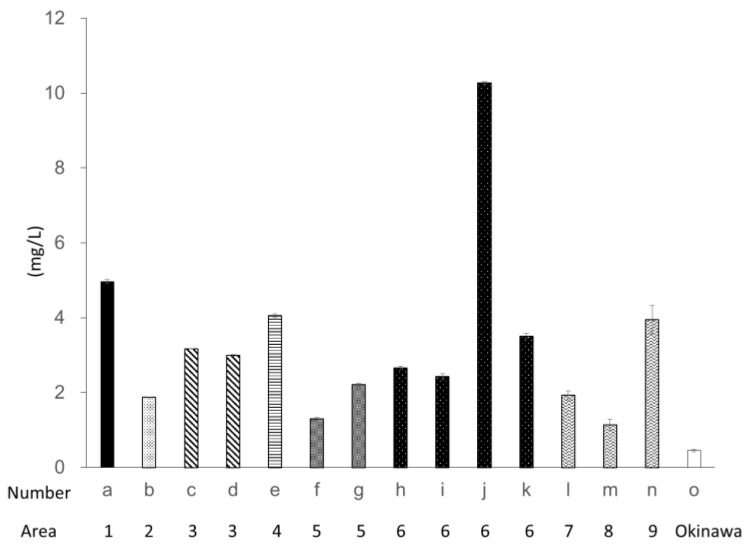
1,1-diphenyl-2-picrylhydrazyl (DPPH) radical scavenging activity of each algal extract. Scavenging activity was expressed as pirogallol solution equivalent. Values are mean ± SE (*n* = 3).

**Figure 3:**
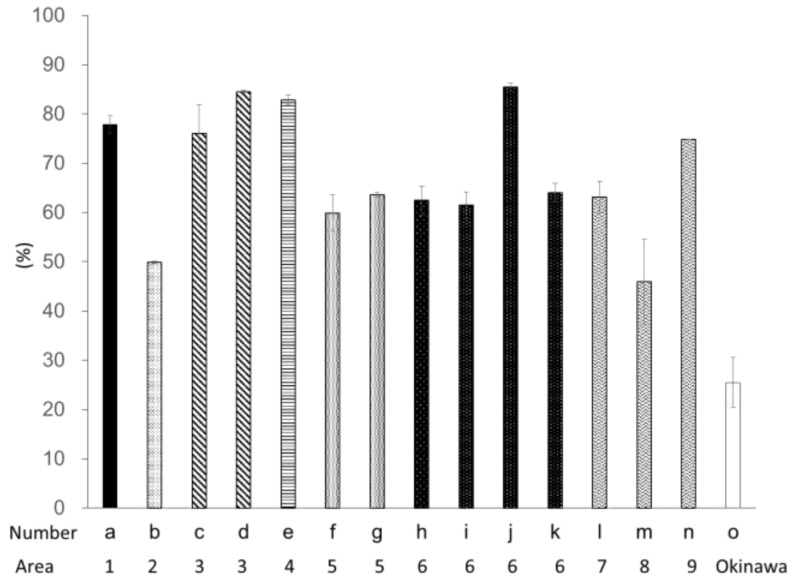
Hydrogen peroxide scavenging activity of each algal extract. Values are mean ± SE (*n* = 3).

The DPPH radical scavenging activities of Ishimozuku are presented in Figure 2. The highest activity was found for sample j. The activity was estimated equal pirogallol solution of 10.3 ± 0.03 mg/L. The average DPPH radical scavenging activity of Ishimozuku was 3.32 ± 0.61 mg/L. Figure 3 shows the hydrogen peroxide scavenging activity of the respective algae extracts. The activity tended to exhibit the same result of DPPH radical scavenging activity. Especially, samples d, e, and j show high anti-oxidative activity in the Ishimozuku sample. Compared to the Ishimozuku extract, the Okinawa-mozuku extract showed low anti-oxidative activity in both experiments.

**Figure 4:**
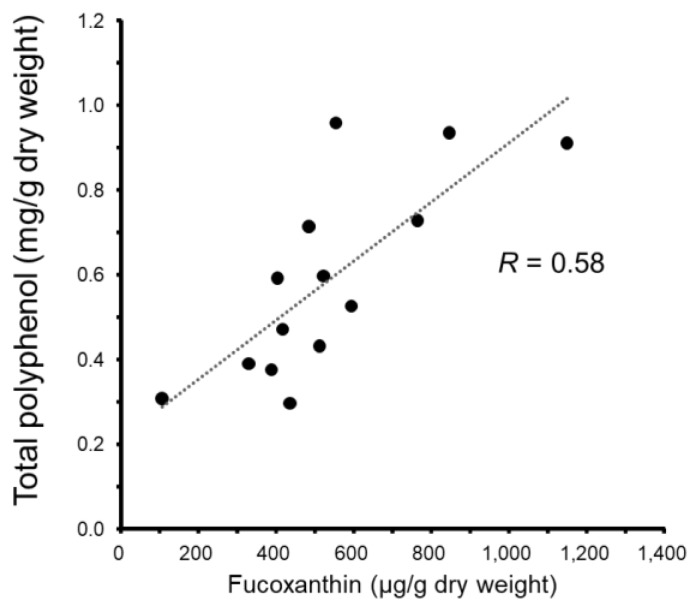
Correlation of total polyphenol and fucoxanthin content of Ishimozuku.

Figure 4 shows correlation between fucoxanthin content and total polyphenol content of Ishimozuku. Their contents were found to have a positive correlation (*R* = 0.58). Results suggest that the high polyphenol contents of Ishimozuku have high fucoxanthin content.

## 3. Discussion

Phenolic compounds are thought to be effective anti-oxidants in brown algae [23,24]. Seaweeds normally have various polyphenols such as gallic acid, catechin, epicatechin, and phlorotannins [25]. These components of bitterness have a function of preventing predation by predatory animals such as sea urchins and shellfish. Furthermore, phlorotannins of the brown seaweed suppress glucose absorption activity considerably by inhibiting their α-glucosidase activity, which is a useful function to inhibit the development of diabetes. In this study, we investigated the polyphenol content of Ishimozuku at several area during several year (Table 1). Results of the present study suggest that harvest year and area differences did not significantly affect the contents. Additionally, the polyphenol content of Ishimozuku samples were almost higher than those of Okinawa-mozuku. Reported phlorotannin contents in several brown algal species are unaffected by the season [26]. Phlorotannins are the major polyphenol components of seaweed. Therefore, it was considered that the polyphenol content of seaweed was more influenced by the difference of species than that of sampling season. From this result, Ishimozuku was considered to be a species with a higher polyphenol content than Okinawa-mozuku. 

Fucoxanthin, a specific pigment of brown algae, shows anti-obesity, anti-cancer, and anti-inflammatory effects. For those reasons, it is used as an ingredient of functional foods [17,18,19,20]. Therefore, high-fucoxanthin content algae are effective not only as a food for improving human health but also as a processed food material such as functional foods. Iwai et al. reported that Okinawa-mozuku contains fucoxanthin in concentrations of about 10–70 µg/g wet weight [27]. The Ishimozuku examined in this study contained about 90.5% water. By calculation, the fucoxanthin contents were 105–737 µg/dry weight. Results show that Okinawa-mozuku contained 153.8 ± 5.80 µg/ g dry weight, and Ishimozuku was 557.9 ± 93.8 µg/g dry weight. The fucoxanthin content of Ishimozuku was higher than that of Okinawa-mozuku in samples in this analysis. 

Polyphenol and fucoxanthin contents showed positive correlation (Figure 4). Results suggest that the high polyphenol contents of Ishimozuku have high fucoxanthin content. Reportedly, fucoxanthin contents of brown algae reach a maximum level in winter (average temperature of seawater is 6~9 °C) [21]. Low-temperature seawater in the north region might upregulate fucoxanthin contents. Reportedly, algal astaxanthin increases because of light stress [28]. Because polyphenols and carotenoids accumulate in plants as a result of the stress response, stress from the environment might be important for fucoxanthin accumulation. Ishimozuku is provided by wild-caught constantly. On the other hands, Okinawa-mozuku is farm-raised seaweed in the south end of Japan. The growth environment may be affecting the accumulation of these components.

Hydrogen peroxide scavenging activity is estimated by quenching hydroxyl radicals produced from hydrogen peroxide catalyzed by oxidized iron. Hydroxyl radicals produced in the body are produced in inflammatory conditions related to several diseases such as cancer, diabetes, and cardiovascular diseases [29]. Although DPPH radical is an artificial chemical, a hydroxyl radical is produced in the body. For that reason, it is useful for analyzing the bioavailability of anti-oxidants of the food materials. The hydrogen peroxide scavenging activities of the respective algal extracts tended to give the same result as that of DPPH radical scavenging activity. Especially, samples d, e, and j showed high hydrogen peroxide scavenging activity in the Ishimozuku sample. Compared with the Ishimozuku extract, the Okinawa-mozuku extract showed low anti-oxidative activity in both experiments. The DPPH radical scavenging activity and hydrogen peroxide scavenging activity are used to estimate water-soluble anti-oxidants. From this result, it was considered that the water-soluble polyphenol components contained abundantly in Ishimozuku showed high anti-oxidant activity. 

The anti-obesity and anti-diabetic effects of fucoxanthin have already been reported by clinical trials. Fucoxanthin was administered to Russian obese women at 2.4 mg/day for 16 weeks [30]. As a result, treated with fucoxanthin group were significantly decreased body weight, body fat, liver fat, plasma triacylglycerol level, and down-regulated the size of the waist circumference. Furthermore, an increase in energy consumption at rest time was confirmed. Li et al. were reported clinical trial of anti-obesity effect by Japanese people. 19 people (male: 12, female: 9) were divided into the placebo group, the fucoxanthin 2 mg/day adoministration group, and the fucoxanthin 4 mg/day administration group, the clinical trial was conducted for 3 months [31]. As a result, the fucoxanthin administration group had significantly reduced body weight and abdominal fat compared to the placebo group. The abdominal fat area was positively correlated with body weight loss. 

Fucoxanthin has been reported to improve glucose tolerance by regulating secretion of adipocytokines in adipocyte cell [32]. Mikami et al. reported clinical trial results on anti-diabetic effect by fucoxanthin administration [33]. Fucoxanthin was administered 1 mg or 2 mg/day for 8 weeks. As a result, hemoglobin A1c level, which is an indicator of the blood glucose level, improved in the group administered with 2 mg. Interestingly, the effect of fucoxanthin was shown to be highly effective in subjects genetically poor in UCP1 expression. From this results, it is considered that fucoxanthin is a highly effective component for people at high risk of obesity.

In this experiment, about 3.58 g on dry weight or 37.7 g on wet weight of Ishimozuku contained 2 mg of fucoxanthin. Ishimozuku is sold in Japan at 70 to 100 g per one portion. Therefore, it is considered to be an amount that can be ingested on a daily basis as a meal. In addition, Ishimozuku also contained polyphenols derived from seaweeds. These polyphenols are expected to exhibit α-glucosidase inhibitory activity and synergistic effects with fucoxanthin function can be expected. It is necessary to further investigate anti-obesity and antidiabetic action by Ishimozuku intake in the future.

Fucoxanthin has been already marketed as a functional food. On the other hand, since fucoxanthin has not been established in large scale chemical synthesis method, it has to be extracted from natural brown alga. Because Ishimozuku is cheaper than seaweed such as Kombu and Okinawa-mozuku, it can be used in the food industry as a food ingredient to obtain fucoxanthin. Moreover, it is thought to be a safe ingredient because it has been eaten by humans.

Furthermore, Ishimozuku has been eaten in processed foods such as vinegar salad in Japan. Seaweed food is low calorie and contain polysaccharides which improve intestinal environment and inhibition of excessive absorption of excess fat and sugar. So, it is popular as a healthy food ingredient. From this study it was shown that Ishimozuku contains a bio-active component which is useful for human health. In recent years consumers have had high functionality needs. Utilization is expected to improve when the health function of Ishimozuku is focused upon in the future. On the other hand, we did not quantify each polyphenol content such as phlorotannins in the present study. Further studies are needed in order to identify and quantify the components of polyphenol or examine bio-activities such as α-glucosidase inhibitory activity and in vivo animal experiments.

## 4. Material and Methods

### 4.1. Material

**Figure 5:**
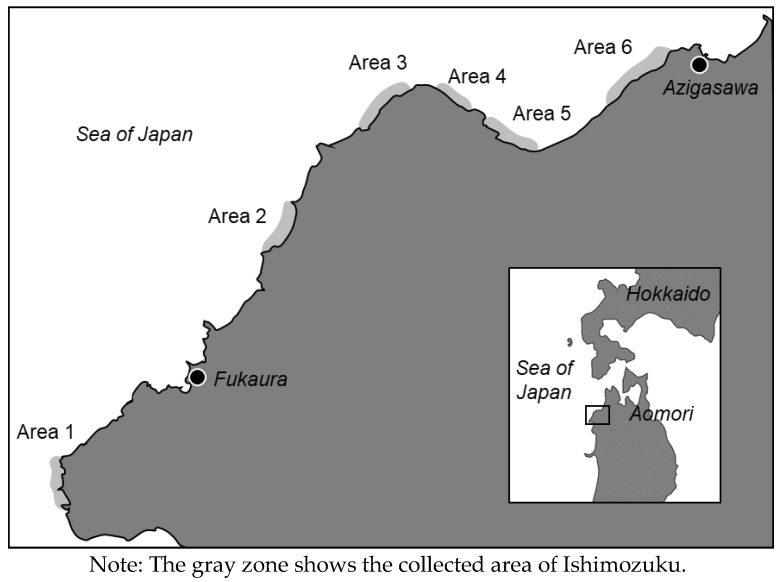
Map of the west coast of Aomori, Japan.

Ishimozuku (*Sphaerotrichia divaricata*) was harvested in the Fukaura town and Azigasawa town Aomori, Japan (Figure 5). Plant samples were collected in 2014 to 2016 during the summer. Okinawa-mozuku (*Cladosiphon okamuranus*) that had been harvested in Okinawa was purchased from a market (Aomori city).

Folin–Ciocalteu’s phenol reagent, DPPH, was purchased from Nacalai Tesque Inc. (Kyoto, Japan). Other chemical reagents were purchased from Wako Pure Chemical Industries Ltd. (Osaka, Japan) or from Nacalai Tesque Inc. (Kyoto, Japan).

### 4.2. Determination of Total Contents of Phenolic Compounds

The total contents of phenolic compounds of the respective algae were found using a modified Folin–Ciocalteu method [25]. Briefly, the fresh algal bodies were freeze-dried before the experiment. The dried bodies (0.2 g) were powdered with a mixer. Then the powder (0.2 g) was extracted with 5 mL of 80% ethanol for 24 h at room temperature. After the supernatant was collected following shaking and centrifugation by 700× *g* for 10 min, the extract was stored at −10 °C until further analysis.

To 0.2 mL of the sample solution, we added 0.4 mL of 10% Folin–Ciocalteu phenol reagent. After 3 min, 0.8 mL of 10% sodium carbonate was added. The mixture was allowed to stand for 30 min. The absorbance was measured at 750 nm by V-730 BIO UV-visible spectrophotometer (JASCO International Co., Ltd., Tokyo, Japan). The phenolic compound contents are expressed as pirogallol equivalent.

### 4.3. Fucoxanthin Quantification by High Performance Liquid Chromatography (HPLC) Analysis

Fucoxanthin contents of extracted samples were analyzed using HPLC according to a modified method reported earlier [21]. In brief, the HPLC system (7000 HPLC system; Hitachi High-Tech Science Corp., Tokyo, Japan) included an L-7000 pump, L-7300 column oven (at 30 °C), and L-7455 diode array detector, which was set to 450 nm. The HPLC mobile phases were methanol/acetonitrile (7:3, *v*/*v*). A reverse-phase column (250 × 4.6 mm, 5.0 μm, Mightysil RP-18GP; Nomura Chemical Co. Ltd., Tokyo, Japan) was used with a guard column (10 × 4.0 mm, 5.0 μm). The fucoxanthin quantity was calculated using a standard curve prepared using purified fucoxanthin.

### 4.4. 1,1-Diphenyl-2-Picrylhydrazyl (DPPH) Radical Scavenging Activity

DPPH radical scavenging activity was found using a method described in an earlier report of the literature [34] with slight modification. Briefly, the extracted solution 0.05 mL were mixed with 0.05 mL of DPPH ethanol solution (400 µM), 0.05 mL of MES buffer (200 mM pH 6.0), and 0.05 mL of ethanol in 96-well microtiter plate. After 30 min, the absorbance was measured at 550 nm by iMark™ microplate absorbance reader (Bio-Rad Laboratories, Inc., Hercules, CA, USA). 

### 4.5. Hydrogen Peroxide Scavenging Activity

Hydrogen peroxide scavenging activity was measured according to the instructions for a commercial kit (Radical catch; Hitachi Ltd., Tokyo, Japan). Briefly, 5 mM of cobalt chloride solution (Reagent A; 50 µL) and luminol solution (Reagent B; 50 µL) were mixed. Then 20 µL of extracted sample solution was added. Subsequently, the solution was incubated at 37 °C for 5 min in an incubator (AccuFLEX Lumi400; Hitachi Ltd., Tokyo, Japan). After the mixture was reacted with hydrogen peroxide solution (Reagent C; 50 µL), we measured the luminescence of light for 120 s in the incubator. The luminescence was observed to subtract an amount of 120 s to 80 s. Control was measured using 80% ethanol instead of extracted sample. Hydrogen scavenging activity was calculated following the equation below.

Hydrogen scavenging activity (%) = {Luminescence (Control)-Luminescence (Sample)}/Luminescence (Control) × 100

### 4.6. Statistical Analysis

Results were expressed as mean ± standard error (SE). Statistical analyses between multiple groups were conducted using analysis of variance (ANOVA). Statistical comparisons were made using Dunnett’s multiple comparison tests. Pearson’s coefficient tests were carried out to test for relationships between total polyphenol and fucoxanthin content of Ishimozuku. Differences were inferred as significant for *p* < 0.05. Analyses were conducted using software (Stat View-J ver. 5.0; SAS Institute Inc., Cary, IL, USA).

## 5. Conclusions

In this study, we provided the first description of the anti-oxidant and fucoxanthin content of Ishimozuku harvested in northern Japan. Our results suggest that Ishimozuku contained several polyphenol compounds and fucoxanthin. The contents were higher than those of Okinawa-mozuku. Ishimozuku is useful for human health for its anti-oxidant activities and fucoxanthin contents.

## Figures and Tables

**Table 1:** Total contents of phenolic compounds and fucoxanthin in dried Ishimozuku (*Sphaerotrichia divaricata*) and Okinawa-mozuku (*Cladosiphon okamuranus*) (nos. a–n is Ishimozuku, no. o is Okinawa-mozuku).

Sample Number	Area Number	Year	Days	Total Polyphenol (mg/g Dry Weight)	Fucoxanthin (µg/g Dry Weight)
a	Area 1	2016	22 June	0.714 ± 0.023 *	483.6 ± 11.9
b	Area 2	2015	7 July	0.308 ± 0.065	105.6 ± 4.60
c	Area 3	2015	2 July	0.597 ± 0.178 *	521.3 ± 94.1 ^#^
d	Area 3	2015	16 September	0.911 ± 0.065 *	1148.5 ± 18.9 ^#^
e	Area 4	2015	9 September	0.727 ± 0.004 *	762.7 ± 7.20 ^#^
f	Area 5	2015	2 July	0.375 ± 0.013 *	387.9 ± 3.30
g	Area 5	2016	7 July	0.390 ± 0.010 *	327.9 ± 3.50
h	Area 6	2015	22 June	0.431 ± 0.011 *	511.1 ± 4.40 ^#^
i	Area 6	2016	24 June	0.296 ± 0.004	435.3 ± 11.03
j	Area 6	2016	19 August	0.958 ± 0.012 *	553.46 ± 12.74 ^#^
k	Area 6	2016	15 September	0.471 ± 0.012 *	415.62 ± 11.03
l	Area 7 A)	2014	30 July	0.592 ± 0.024 *	403.1 ± 40.7
m	Area 8 A)	2014	30 July	0.525 ± 0.048 *	593.2 ± 101 ^#^
n	Area 9 A)	2014	30 July	0.935 ± 0.075 *	845.0 ± 74.2 ^#^
o	Okinawa, Japan	2014	July	0.082 ± 0.011	153.8 ± 5.80

Values are mean ± standard error (SE) (*n* = 3). A) It was harvested in Fukaura area. However, the detail is unclear. * *p* < 0.05 vs. Okinawa-mozuku of Total polyphenol ^#^ *p* < 0.05 vs. Okinawa-mozuku of fucoxanthin.
